# Prevalence and co-use of marijuana among young adult cigarette smokers: An anonymous online national survey

**DOI:** 10.1186/1940-0640-7-5

**Published:** 2012-04-19

**Authors:** Danielle E Ramo, Judith J Prochaska

**Affiliations:** 1 Department of Psychiatry, University of California at San Francisco, 401 Parnassus Avenue,, Box TRC 0984, San Francisco, CA 94143, USA

**Keywords:** Marijuana, Tobacco, Young adults, Internet

## Abstract

**Background:**

There is elevated prevalence of marijuana use among young adults who use tobacco, but little is known about the extent of co-use generated from surveys conducted online. The purpose of the present study was to examine past-month marijuana use and the co-use of marijuana and tobacco in a convenience sample of young adult smokers with national US coverage.

**Methods:**

Young adults age 18 to 25 who had smoked at least one cigarette in the past 30 days were recruited online between 4/1/09 and 12/31/10 to participate in an online survey on tobacco use. We examined past 30 day marijuana use, frequency of marijuana use, and proportion of days co-using tobacco and marijuana by demographic characteristics and daily smoking status.

**Results:**

Of 3512 eligible and valid survey responses, 1808 (51.5%) smokers completed the survey. More than half (53%, n = 960) of the sample reported past-month marijuana use and reported a median use of 18 out of the past 30 days (interquartile range [IR] = 4, 30). Co-use of tobacco and marijuana occurred on nearly half (median = 45.5%; IR = 13.1, 90.3) of the days on which either substance was used and was more frequent among Caucasians, respondents living in the Northeast or in rural areas, in nonstudents versus students, and in daily versus nondaily smokers. Residence in a state with legalized medical marijuana was unrelated to co-use or even the prevalence of marijuana use in this sample. Age and household income also were unrelated to co-use of tobacco and marijuana.

**Conclusion:**

These results indicate a higher prevalence of marijuana use and co-use of tobacco in young adult smokers than is reported in nationally representative surveys. Cessation treatments for young adult smokers should consider broadening intervention targets to include marijuana.

## Background

Epidemiologic data indicate US young adult smokers use marijuana in greater amounts that their non-smoking peers. In 2009, 34.6% of smokers aged 18 to 25 reported past-month cannabis use compared with 8.9% of young adult nonsmokers [1]. Depending on definitions of use, tobacco use increases the risk of cannabis use from 2 (e.g., past 30-day tobacco use is associated with past 30-day marijuana use [2]) to 52 times (e.g., having ever tried tobacco is associated with having ever tried marijuana [3]) in adolescents, and 3 to 6.4 times in adults [4-6].

Demographic differences have been observed in patterns of tobacco and marijuana involvement among young adults. Older youths [7,8], males [6,8-10], students in vocational schools [7], and those living in the Northeast and in small metropolitan areas [11,12] are more likely to use tobacco or cannabis. There is a need to examine more detailed patterns of tobacco and marijuana use to understand the complex relationship between these two substances.

The internet is increasingly used in survey research of substance use [13,14] with benefits over face-to-face interviews including broader reach; greater inclusion of low-incidence or “hidden” populations; rapid, convenient input by respondents; and reduced bias in response to sensitive, potentially stigmatizing topics including illicit substance use [15-18]. Young adults remain the age group most likely to use the internet (93% in a recent survey [19]), and they are less likely, compared to other age groups, to present to traditional research settings for studies of health behavior [20,21]. Our prior research has demonstrated the reliability and validity of anonymous online surveys of young adult tobacco and cannabis use [22,23].

Analyzing data from an anonymous online survey of young adult smokers with national coverage, the present study examined the prevalence of past-month marijuana use, frequency (days of use) among past-month marijuana users, and the frequency of co-using tobacco and marijuana. The large sample permitted analyses by gender, age, ethnicity, geographic region, urban/rural designation, student status, household income, daily smoking status, and by whether or not respondents resided in a state where marijuana is legal for medicinal use.

## Methods

Data for the present study were taken from a national cross-sectional survey using a convenience sample of young adult smokers. Recruitment methods and survey design have been described in detail previously [22,24]. Briefly, young adults between the ages of 18 and 25, who reported smoking at least one cigarette in the past 30 days, were recruited online between 4/1/09 and 12/31/10. Three recruitment methods were used: 1) a paid advertisement campaign on Facebook; 2) a free campaign on Craigslist; and 3) a paid email advertising campaign through a survey sampling company. Participant entries could be tracked to which advertisement type they viewed (i.e., those targeting tobacco only, n = 6423; or those targeting tobacco and marijuana use, n = 7567). Only entries from advertisements targeting tobacco use were used in the present study so as not to inflate the prevalence of marijuana use in this population. Advertisements invited young adults to participate in a 20-minute online survey on tobacco use (with no reference to marijuana) with a chance to win a prize in a drawing worth either US $25 or $400. Advertisements contained a hyperlink directing potential participants to the study’s institutional review board (IRB)-approved consent form, which mentioned assessment of marijuana use; to a screener for eligibility criteria; and to a secure online survey with data encryption for added security. Computer IP addresses were tracked, and only one entry was allowed from a single computer to prevent duplicate entries from the same person; however, multiple entries were allowed from the same internet connection (e.g., dormitories, apartment buildings).

## Measures

### Substance use

Presence or absence of past 30-day tobacco use was assessed using a single screening item: “Have you smoked at least one cigarette in the past month (30 days)? [y/n].” This item was corroborated with data from the Smoking Questionnaire [25] and Timeline Followback [26], and only responses that were consistent across the three measures were used in the present study. Daily smokers were categorized as those who indicated they smoked, on average, 7 days a week on the Smoking Questionnaire. Cannabis use was assessed with the Timeline Followback method [26], from which past 30-day use [y/n], number of days using in the past 30 days, and percent of days using both tobacco and marijuana out of total days using either substance were calculated.

### Sociodemographics

Gender, age, race/ethnicity, student status, and annual family income were assessed. Residential zip codes were used to categorize participants as residing in: 1) one of four US Census Regions (Northeast, Midwest, South, and West) [27]; 2) an urban or rural area (using zip code approximations of rural–urban communing area data from the 2000 census in a coding system made public by the University of Washington Rural Health Research Center) [28]; and 3) one of the 16 states or Washington, DC, in which there was an active medical marijuana program at the time of data collection.

### Statistical analyses

Analyses were restricted to completed surveys (N = 1808) and examined marijuana prevalence (% of sample using), days using marijuana in the past 30 days (among marijuana users), and percentage of days co-using tobacco and marijuana. Due to problems with skew and kurtosis in reported frequency of marijuana use and percent of days co-using, nonparametric statistical tests (Mann–Whitney for two-group tests or Kruskal-Wallis for >2 group tests) were used to examine differences in marijuana-use characteristics by demographic variables and daily smoking status. Analyses of marijuana use by gender were limited to only those participants who identified as male or female due to the small number of transgender participants (n = 8).

## Results

During the recruitment period, the online survey received more than 6423 hits, and 6176 people gave online consent to determine eligibility; of these, 3512 (56.9%) were eligible and deemed to be valid cases. Of eligible and valid cases, 2998 (85.4%) completed information about demographic and tobacco use only, and 1808 (51.5%) completed the entire 20–30 minute survey. Those who completed the survey (n = 1808) differed from those who didn’t (n = 1190) on some demographic variables, but the differences were small (i.e., the completer group was 64% male with a mean age of 20.5 years and 13.0 years of education, while the noncompleter group was 69% male with a mean age of 20.1 years and 12.8 years of education).

The majority of the sample was male (64%), Caucasian (72%), living in an urban area (85%), not currently a student (71%), and smoked marijuana daily (68%) (Table 1). Among current smokers, the overall prevalence of marijuana use was 53%. There was a significantly higher prevalence of marijuana use among males compared with females; among those aged 18 to 20 compared with those aged 21 to 25; among those with higher household income; among those living in urban versus rural areas; and among nondaily versus daily smokers. There were no differences in prevalence of recent cannabis use by ethnicity, census region, residence in a medical marijuana state, or student status.

**Table 1:** Prevalence of Marijuana Use among Young Adults Who Use Both Tobacco and Marijuana

**Variable**	**Percentage of Sample (*n*)**	**Percentage with Past-Month** **Marijuana Use**	***χ***^**2**^	**p**
Gender*			**8.69**	**0.003**
Male	64.3 (1162)	55.7		
Female	35.3 (638)	48.4		
Transgender	0.4 (8)	50.0		
Age			**27.82**	**0.000**
18–20	56.2 (1016)	58.6		
21–25	43.8 (792)	46.1		
Ethnicity			10.59	0.060
African-American	2.9 (52)	53.8		
Asian-American/Pacific Islander	3.8 (69)	39.1		
Caucasian-American	72.1 (1302)	52.8		
Hispanic/Latino	5.8 (105)	56.2		
Multi-Ethnic	9.7 (176)	60.8		
Other	5.7 (103)	49.5		
Household Income			**15.20**	**0.020**
Less than $20,000	26.1 (472)	48.7		
$21,000–$40,000	21.5 (388)	50.0		
$41,000–$60,000	15.3 (276)	53.3		
$61,000–$80,000	10.8 (195)	53.8		
$81,000–$100,000	8.6 (156)	58.3		
$100,000–$200,000	11.9 (216)	57.4		
More than $200,000	5.8 (105)	65.7		
Region			1.14	0.769
Northeast	20.1 (363)	52.1		
Midwest	26.5 (479)	52.4		
South	28.0 (506)	52.6		
West	25.4 (460)	55.2		
Urban/Rural			**8.75**	**0.003**
Urban	85.2 (1540)	54.5		
Rural	14.8 (268)	44.8		
Medical Marijuana State			2.02	0.155
Yes	35.8 (647)	55.3		
No	64.2 (1161)	51.9		
Student Status			0.44	0.508
Student	28.8 (521)	54.3		
Non-student	71.2 (1287)	52.6		
Daily Smoking Status			**4.00**	**0.045**
Daily smoker	68.3 (1235)	51.5		
Non-daily smoker	31.7 (573)	56.5		
Total Sample		53.1		

*Note.* Significant differences within sociodemographic variables in bold text.

*Eight transgendered participants were not included in gender analysis of past-month marijuana use.

Among past-month marijuana users, the median number of days using marijuana was 18.0 (IR = 4, 30) in the past 30 days (Table 2). Nonstudents used marijuana on significantly more days than students, and daily smokers used on significantly more days than nondaily smokers. There were no differences in the number of days using marijuana in the past month by gender, age, ethnicity, household income, region, urban versus rural residence, or residence in a medical marijuana state.

**Table 2:** Past 30-Day Marijuana Use and Tobacco and Marijuana Co-Use among Past-Month Marijuana Users (n = 960) by Sociodemographic Characteristics

	**Past 30-Day Marijuana Use**				**Percentage of Days Using Tobacco or Marijuana in which Both Substances Were Used**			
Variable	Median	Interquartile Range	Group Comparisons	*p*	Median	Interquartile Range	Group comparisons	*p*
Gender			z = -1.01	0.311			z = -.25	0.801
Male	19.0	(5.0, 30.0)			48.4	(13.8, 90.3)		
Female	16.0	(4.0, 30.0)			42.1	(12.9, 90.3)		
Age			z = -1.10	0.271			z = -.54	0.588
18–20	18.0	(5.0, 30.0)			48.0	(14.3, 89.3)		
21–25	17.0	(3.0, 30.0)			45.2	(10.9, 93.5)		
Ethnicity			*χ*_(5)_^2^ = 5.78	0.300			***χ***^**2**^_**(5)**_ **= 11.22**	**0.047**
African-American	8.0	(4.0, 28.5)			35.1	(13.0, 72.4)		
Asian/Pacific Islander	17.0	(3.0, 30.0)			14.3	(6.3, 74.2)		
Caucasian	18.5	(5.0, 30.0)			50.0	(14.3, 93.5)		
Hispanic/Latino	15.0	(2.0, 26.0)			38.7	(6.5, 78.6)		
Multi-Ethnic	17.0	(4.0, 30.0)			42.1	(13.0, 83.3)		
Other	14.0	(6.0, 30.0)			41.9	(14.3, 93.5)		
Household Income			*χ*_(6)_^2^ = 2.96	0.814			*χ*_(6)_^2^ = 4.71	0.582
Less than $20,000	16.0	(5.0, 30.0)			47.8	(14.7, 90.3)		
$21,000–$40,000	19.0	(5.0, 30.3)			52.7	(12.5, 94.3)		
$41,000–$60,000	20.0	(3.0, 30.0)			51.6	(12.5, 96.8)		
$61,000–$80,000	17.0	(4.0, 29.0)			40.0	(14.0, 82.3)		
$81,000–$100,000	18.0	(4.0, 30.0)			48.4	(12.9, 96.8)		
$100,000–$200,000	18.0	(6.3, 28.0)			41.8	(13.1, 76.8)		
Region			*χ*_(3)_^2^ = 1.83	0.609			***χ***^**2**^_**(3)**_ **= 11.12**	**0.011**
Northeast	19.0	(7.0, 30.0)			57.1	(19.7, 93.5)		
Midwest	17.0	(7.0, 30.0)			48.4	(22.2, 90.3)		
South	16.0	(4.0, 30.0)			42.0	(12.8, 84.1)		
West	19.0	(3.8, 30.0)			38.3	(9.1, 90.3)		
Urban/Rural			z = -1.48	0.140			**z = -2.35**	**0.019**
Urban	17.0	(4.0, 30.0)			45.2	(12.9, 87.1)		
Rural	20.5	(6.25, 31.0)			57.9	(22.6, 96.8)		
Medical Marijuana State			z = -0.42	0.672			z = -1.10	0.272
Yes	19.0	(4.0, 30.0)			41.7	(10.7, 93.5)		
No	17.0	(4.0, 30.0)			48.3	(15.9, 87.3)		
Student Status			**z = -4.00**	**0.000**			**z = -4.59**	**0.000**
Student	13.0	(3.0, 28.0)			30.0	(7.1, 77.4)		
Non-student	19.0	(6.0, 30.0)			51.6	(17.0, 93.5)		
Daily Smoking Status			**z = -5.34**	**0.000**			**z = -11.49**	**0.000**
Daily smoker	21.0	(7.0, 30.0)			65.6	(22.7, 96.8)		
Non-daily smoker	10.5	(3.0, 26.0)			20.0	(5.6, 47.2)		
Total Sample	18.0	(4.0, 30.0)			45.5	(13.1, 90.3)		

*Note.* Tests of group differences with two groups used Mann–Whitney z-test, and more than two groups used Kruskal-Wallace chi-square (*χ*^2^). Significant differences within sociodemographic variables are in bold text.

The proportion of days using both substances out of all past-month using days was a median of 45.5% (IR = 13.1, 90.3). There was a higher proportion of tobacco and marijuana co-use among Caucasian respondents compared with those of other ethnic groups, among those residing in the Northeast compared to other census regions, among those residing in rural versus urban areas, among nonstudents, and among daily versus nondaily smokers (Table 2). There were no differences in percentage of days with co-use by gender, age, household income, or residence in a medical marijuana state.

## Discussion

The findings from this online anonymous survey of young adult smokers with national coverage indicate a greater prevalence of marijuana use than has been reported in epidemiological studies using household interviews. For example, in 2009, the US Substance Abuse and Mental Health Services Administration (SAMHSA) National Survey on Drug Use and Health [1] reported that 34.6% of past-month smokers age 18 to 25 used marijuana, compared with 53.1% reported in the present study. The present sample was recruited online, primarily through social media, and the survey was completely anonymously, potentially allowing for reduced bias in reporting of illegal substance use (i.e., marijuana use).

High prevalence of use was observed across demographic groups and regions, suggesting the issue of marijuana and tobacco co-use is of national relevance. The highest prevalence of marijuana use was observed among males, younger people, those with a higher household income and living in urban areas, and nondaily tobacco smokers. Consistent with previous epidemiological studies, young adult males tended to use marijuana at higher levels than young adult females [29], and young adults tended to reduce substance use as they reached developmental milestones of emerging adulthood, including leaving home, obtaining stable employment, and starting a family [30]. Greater use among those in urban areas and from wealthier households reflects factors related to availability and is also consistent with national trends from household survey data [31].

Notably, although daily tobacco smokers were slightly less likely to use marijuana than nondaily smokers, when they did use, they used it more frequently. There was a two-fold greater frequency of use among daily smokers compared with nondaily smokers and elevated frequency of use among nonstudents. Nonstudents and daily smokers also had greater co-use. Given the potential for detrimental effects of co-use among daily smokers, these findings support the broadening of interventions for daily tobacco smokers to consider use of both substances. Future research should examine the potential for substitution or compensatory effects during attempts to quit either substance [32].

Study limitations include convenience sampling and self-reported data; however, face-to-face surveys often similarly rely on self-reported drug use, and we have previously demonstrated strong reliability and validity of tobacco [22] and marijuana [23,33] online surveys with young adults. The survey completion rate in this study was comparable to online survey studies with young adults [34] but lower than that typically seen in nationally representative surveys. For example, weighted response rates for the 2010 SAMHSA-sponsored National Survey on Drug Use and Health were 88.8% for household screening and 74.7% for household interviewing [35]. Our respondents could leave the survey at any time; methods considered to encourage completion would have compromised participant anonymity. Sampling procedures and online data collection could have led to higher prevalence of marijuana use and co-use than is typical of representative surveys that have procedures to increase response rates (e.g., mailings, phone calls, household visits).

## Conclusion

The current findings indicate that tobacco and marijuana co-use is common. The significant public-health effects of tobacco and marijuana use have been well-documented [36-39]. Cessation treatments for young adult smokers should consider broadening intervention targets to include marijuana, and conversely, those for marijuana should include tobacco. With increasing use of the internet for optimizing reach to young adults for health behavior change research [13,14,40], the online medium will likely be instrumental in helping to understand and treat multiple substance use in young adults.

## Competing interests

The authors declare that they have no competing interests.

## Authors’ contributions

Dr. Ramo designed the study and wrote the protocol in consultation with Dr. Prochaska. Dr. Ramo carried out the survey study with mentorship from Dr. Prochaska. Dr. Ramo completed the first draft of the manuscript, and Dr. Prochaska reviewed and revised subsequent drafts. Both authors contributed to and have approved the final manuscript.
